# Improved procedural workflow for catheter ablation of paroxysmal AF with high‐density mapping system and advanced technology: Rationale and study design of a multicenter international study

**DOI:** 10.1002/clc.23806

**Published:** 2022-04-21

**Authors:** Francesco Solimene, Giuseppe Stabile, Pablo Ramos, Luca Segreti, Filippo Maria Cauti, Valerio De Sanctis, Ruggero Maggio, Javier Ramos‐Maqueda, Lluis Mont, Vincenzo Schillaci, Maurizio Malacrida, Ignacio Garcia‐Bolao

**Affiliations:** ^1^ Clinica Montevergine Mercogliano Italy; ^2^ Department of Cardiology and Cardiovascular Surgery Clìnica Universidad de Navarra Pamplona Spain; ^3^ IdiSNA, Navarra Institute for Health Research Navarra Spain; ^4^ Second Division of Cardiology, Cardiac‐Thoracic‐Vascular Department New Santa Chiara Hospital, Azienda Ospedaliero Universitaria Pisana Pisa Italy; ^5^ Arrhythmology Unit, Ospedale San Giovanni Calibita, Fatebefratelli, Isola Tiberina Rome Italy; ^6^ Istituto Clinico Sant'Ambrogio Milan Italy; ^7^ Infermi Hospital Rivoli Italy; ^8^ Department of Cardiology Section for Electrophysiology, Lozano Blesa Clinical University Hospital Zaragoza Spain; ^9^ Hospital Clínic University of Barcelona Barcelona Spain; ^10^ Boston Scientific, Scientific Communications Milano Italy

**Keywords:** antral potential, atrial fibrillation, catheter ablation, high‐density mapping system, local impedance, Lumipoint^TM^

## Abstract

**Background:**

The antral region of pulmonary veins (PV)s seems to play a key role in a strategy aimed at preventing atrial fibrillation (AF) recurrence. Particularly, low‐voltage activity in tissue such as the PV antra and residual potential within the antral scar likely represent vulnerabilities in antral lesion sets, and ablation of these targets seems to improve freedom from AF. The aim of this study is to validate a structured application of an approach that includes the complete abolition of any antral potential achieving electrical quiescence in antral regions.

**Methods:**

The improveD procEdural workfLow for cathETEr ablation of paroxysmal AF with high density mapping system and advanced technology (DELETE AF) study is a prospective, single‐arm, international post‐market cohort study designed to demonstrate a low rate of clinical atrial arrhythmias recurrence with an improved procedural workflow for catheter ablation of paroxysmal AF, using the most advanced point‐by‐point RF ablation technology in a multicenter setting. About 300 consecutive patients with standard indications for AF ablation will be enrolled in this study. Post‐ablation, all patients will be monitored with ambulatory event monitoring, starting within 30 days post‐ablation to proactively detect and manage any recurrences within the 90‐day blanking period, as well as Holter monitoring at 3, 6, 9, and 12 months post‐ablation. Healthcare resource utilization, clinical data, complications, patients' medical complaints related to the ablation procedure and patient's reported outcome measures will be prospectively traced and evaluated.

**Discussion:**

The DELETE AF trial will provide additional knowledge on long‐term outcome following a structured ablation workflow, with high density mapping, advanced algorithms and local impedance technology, in an international multicentric fashion. DELETE AF is registered at ClinicalTrials.gov (NCT05005143).

## 1 INTRODUCTION

The standard approach to the treatment of paroxysmal atrial fibrillation (PAF) is pulmonary vein isolation (PVI).^1^, ^2^ Complete electrical isolation of the PVs with documented entrance and/or exit block has better rhythm outcomes than incomplete isolation;^3^ however, long‐term atrial arrhythmia‐free survival is still unsatisfactory^4^ and a high incidence of PV reconnection has been reported in patients with clinical recurrences of AF.^2^, ^5^, ^6^ The antral region of PVs seems to play a key role in a strategy aimed at preventing AF recurrence.^7^ Particularly, low‐voltage activity in tissues such as the PV antra and residual potential within the antral scar (RAP) likely represent vulnerabilities in antral lesion sets, and ablation of these targets seems to improve freedom from AF as reported by Segerson et al.^8^ in a single‐center fashion by using the Rhythmia^TM^ HDx mapping system (Rhythmia^TM^; Boston Scientific) and standard radiofrequency (RF) ablation catheters. These results were driven by performing high‐density mapping of PV antra after traditional encirclement lesions through a structured workflow approach. Broader clinical application of this technique and recreation of these high AF efficacy rates await confirmation by other investigators and validation with a prospective, multicenter large clinical study, also by using the most advanced mapping and ablative technologies. The aim of the improveD procEdural workfLow for cathETEr ablation of PAF with high‐density mapping system and advanced technology (DELETE AF) study is to fill this clinical evidence gap.

## 2 METHODS

The study complied with the Declaration of Helsinki, the locally appointed ethics committee approved the research protocol, and informed consent will be obtained from all patients. This trial is registered at clinicaltrials. gov (NCT05005143).

### 2.1 Study design

**Figure 1:**
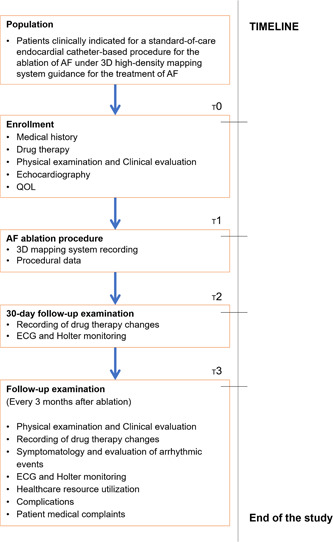
DELETE AF study flowchart. 3D, three dimensional; DELETE AF, improveD procEdural workfLow for cathETEr ablation of paroxysmal atrial fibrillation; ECG, electrocardiogram; QOL, quality of life

DELETE AF is a prospective, single‐arm, international postmarket cohort study designed to demonstrate a low rate of clinical atrial arrhythmias recurrence with an improved procedural workflow for catheter ablation of PAF, using the most advanced point‐by‐point RF ablation technology in a multicenter setting. The study flowchart is depicted in Figure 1. The study is approved by the Institutional Review Boards of the participating centers. The trial will be conducted in accordance with the “Declaration of Helsinki” (1964 and later amendments).

### 2.2 Patient enrollment

Enrollment will start in January 2022 and are planned to end in December 2022. Up to 300 consecutive patients are planned to be enrolled at 10 European centers. All patients will undergo a baseline screening examination, to verify eligibility criteria. After signing an informed consent form, consecutive patients undergoing AF ablation will be prospectively enrolled. The patient's clinical data and procedural management information will be collected.

### 2.3 Patient population

Subjects enrolled in the DELETE AF study will be clinically indicated for a standard‐of‐care endocardial catheter‐based ablation procedure under the Rhythmia^TM^ mapping system guidance for the treatment of AF and meet the study inclusion/exclusion criteria as outlined in Table 1.

**Table 1:** Inclusion and exclusion criteria for enrollment in the DELETE AF study

Inclusion criteria: a) History of recurrent symptomatic PAF with ≥1 episode reported and documented within the 365 days before enrollment b) Indication to an ablation procedure with 3D high‐density mapping system according to current international and local guidelines c) Willingness and ability to provide informed consent, participating in all testing at an approved clinical investigational center d) Age ≥18 years or above, and of legal age to give informed consent specific to state and national law
Exclusion criteria: a) Persistent or long‐standing persistent AF b) Participation in another clinical study that may have an impact on the study endpoints c) Unwillingness or inability to sign an authorization to use and disclose health information or an Informed Consent form d) Unwillingness or inability to complete follow‐up visits and examination for the duration of the study at the center e) Life expectancy ≤12 months f) Previous cardiac ablation within 90 days before enrollment g) Unrecovered/unresolved adverse events from any previous invasive procedure h) Women of childbearing potential who are, or plan to become, pregnant during the time of study i) Left atrial size >60 mm diameter on echocardiogram j) Left atrial thrombus in preprocedure imaging within 4 weeks of the ablation procedure k) AF secondary to electrolyte imbalance, thyroid disease, or reversible noncardiac cause l) History of prior surgical ablation for AF or atypical atrial flutter, including MAZE or mini‐MAZE

Abbreviations: DELETE AF, improveD procEdural workfLow for cathETEr ablation of paroxysmal atrial fibrillation; PAF, paroxysmal atrial fibrillation.

### 2.4 Ablation treatment

All procedures will be performed under conscious sedation or general anesthesia according to centers' preference. All patients will be treated according to a prespecified structured workflow followed by each center. After single or double transseptal punctures under fluoroscopic guidance, intravenous unfractionated heparin boluses will be administered, to maintain an activated clotting time of >300 seconds. The basket mapping catheter (Orion^TM^; Boston Scientific) and the ablation catheter (IntellaNav MiFi OI^TM^ or IntellaNav StablePoint OI^TM^; Boston Scientific) will be then inserted through steerable sheaths. The Orion^TM^ catheter will be used in combination with the Rhythmia^TM^ HDx mapping system to create a three‐dimensional electroanatomical voltage and activation map of the left atrium. The ablation target in all patients will be PVI and the achievement of complete electrical quiescence within the antral region. PVI will be performed by means of RF energy in a point‐by‐point ablation pattern by the open‐irrigated ablation catheter. Point‐by‐point RF delivery will be performed in such a way as to create contiguous ablation spots encircling the PVs. The maximal interlesion distance between two neighboring lesions will be set ≤6 mm. Ablation will be guided by the magnitude and time course of the impedance drop during RF delivery.^9^, ^10^, ^11^ Power settings will be set at the individual operator's discretion, within the range of 30–50 W, depending on the left atrial segment considered.

**Figure 2:**
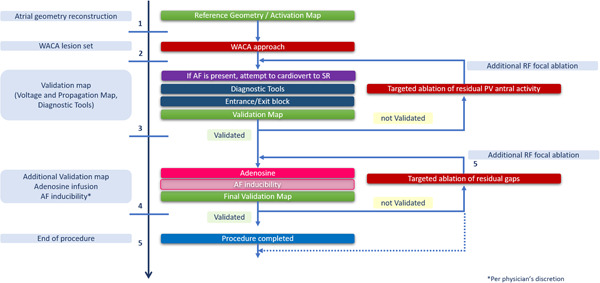
DELETE AF procedural workflow. DELETE AF, improveD procEdural workfLow for cathETEr ablation of paroxysmal atrial fibrillation; PV, pulmonary vein; RF, radiofrequency

**Figure 3:**
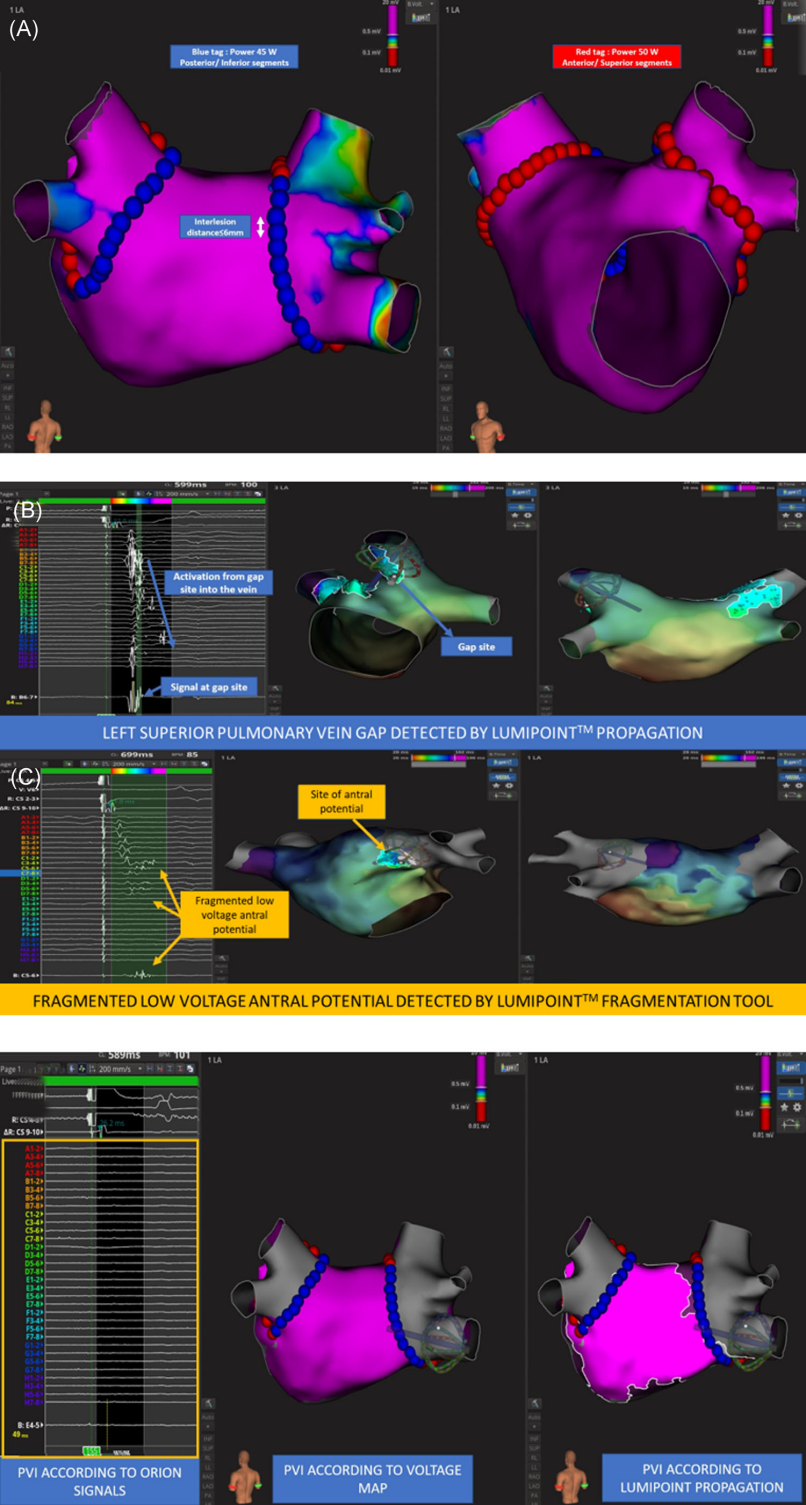
(A) The DELETE AF ablation protocol. Posterior view (left panel) and anterior view (right panel), using the Rhythmia^TM^ mapping system. Point‐by‐point RF delivery created contiguous ablation spots encircling the PVs. The maximal interlesion distance between two neighboring lesions was set ≤6 mm and was automatically measured through the Autotag^TM^ software. Power settings are set at the individual operator's discretion, within the range of 30–50 W, depending on the left atrial segment considered. In this example, power setting was 45 W at the posterior site (blue dots) and 50 W at anterior sites (red dots). (B) Examples of PVG (upper panel) and residual RAP (lower panel) detected through Lumipoint^TM^ tool according to the procedural workflow. (C) After the antral lesion set was delivered, the Orion^TM^ catheter was engaged in the PVs to assess circumferential luminal electrical activity and the Lumipoint^TM^ tool was used sequentially on each PV component, to assess the presence of PVGs and RAPs and to characterize electrical propagation. In this example, antral isolation and electrical quiet were demonstrated in all the PVs after PV isolation and antral ablation. DELETE AF, improveD procEdural workfLow for cathETEr ablation of paroxysmal atrial fibrillation; PV, pulmonary vein; PVG, PV gap; RAP, residual potential within the antral scar; RF, radiofrequency

The endpoint of ablation is PVI, as assessed on the basis of entry and exit block by means of the 64‐pole Orion^TM^ catheter placed sequentially in each of the PVs, and the achievement of complete electrical quiescence within the antral region. The Lumipoint^TM^ map analysis tool, which automatically identifies fragmented potentials and continuous activation, will be used sequentially on each PV component, to assess the presence of PV gaps (PVGs) and RAPs and to characterize electrical propagation. After identification of an atrial site with PVG or RAP by means of the Lumipoint^TM^ software, both the voltage map and the standard activation/propagation map will be investigated to check for agreement. Regarding the ablation strategy, each residual PVG will be first targeted to guarantee contiguity in the lesion set. Second, after confirmation of bidirectional isolation, when any low‐voltage propagation is suspected within the region of previous antral ablation and are confirmed by information from the Lumipoint^TM^ tool and by consistency with regional activation timing,^8^ additional focal ablations will be performed. These ablations will be then reassessed by means of repeat Lumipoint^TM^ and voltage and propagation mapping until electrical quiescence will be achieved. A detailed description of the procedural workflow is depicted in Figure 2. Example of the ablation workflow, identification of PVGs and residual RAPs detected through Lumipoint^TM^ according to the procedural workflow and demonstration of PVI, and complete electrical quiescence at the end of the ablation are reported in Figure 3A–C.

### 2.5 Follow‐up evaluation program

Post ablation, all patients will be monitored with ambulatory event monitoring, starting within 30 days post ablation to proactively detect and manage any recurrences within the 90‐day blanking period, as well as Holter monitoring at 3, 6, 9, and 12 months post ablation. Additional electrocardiogram (ECG) monitoring will be performed as indicated by patient symptoms. At each visit, a medical history will be performed to ascertain if there has been a change in medications or of cardiac status (Figure 1). Healthcare resource utilization (hospital or emergency room access, planned and/or unplanned follow‐up visits, diagnostic examinations), clinical data, complications, and patients' medical complaints related to the ablation procedure will be prospectively traced. Patient‐reported outcomes questionnaires will be completed at each study visit to measure the quality of life: the EQ‐5D‐5L, the Specific Symptom Scale Score,^12^, ^13^, ^14^ and the EHRA Score of AF‐related Symptoms.

### 2.6 Study hypotheses

This prospective cohort study has been specifically designed to demonstrate a low rate of clinical atrial arrhythmia recurrence with an improved procedural PVI workflow^8^ for catheter ablation of PAF in a multicenter setting, using the most advanced point‐by‐point technology versus recent literature reference on ablation of PAF.^15^, ^16^, ^17^, ^18^

Apart from its primary purpose, the study will evaluate the association between the occurrence of atrial arrhythmias and baseline patient's characteristics (clinical history and drug therapy) or procedural data (post hoc voltage and activation maps analysis), will estimate costs associated with the use of health care resources, rate of the adverse events associated with the primary ablation procedure, and overall procedure time. Moreover, periprocedural factors (e.g., periprocedural anticoagulation management or total fluoroscopy time and contrast medium utilization) associated with the 12‐month outcome and the acute procedural success will be investigated.

### 2.7 Primary endpoint

The primary endpoint of the study is the rate of success at the medium‐term follow‐up after PVI in consecutive patients undergoing AF ablation. Specifically, the primary outcome measure of this study is the percentage of patients with documented arrhythmias after 12 months from the procedure. Arrhythmias are defined as any AF, atrial flutter, or atrial tachycardia episode, continuously for >30 seconds, as recorded by any postablation ECG modality. Arrhythmia recurrences within the first 3 months (blanking period) are classified as early recurrences and will not be considered a procedural failure.

### 2.8 Secondary endpoints

Nine secondary endpoints will be evaluated. Specifically:

1. Acute procedural success of AF ablation. The acute success of the AF ablation is verified by the completion of the necessary ablation application(s), the validation of ablation through appropriate technique(s), and, when applicable, the termination of the primary arrhythmia.
2. Recurrence of AF‐only arrhythmia after 12 months from the procedure.
3. Occurrence of all kinds of atrial arrhythmias. A sustained atrial flutter/atrial tachycardia is defined as >30 seconds arrhythmia occurrence as recorded on a monitoring device.
4. Percentage of patients free from any clinical atrial arrhythmias according to procedure type (de novo vs. redo ablation).
5. Association between baseline patient's characteristics and electroanatomical variables derived from the evaluation of voltage and activation maps and arrhythmias' occurrence at 12 months.
6. Repeated ablation procedures (after the index procedure). The number of ablation procedures for the treatment of the primary arrhythmia after the index procedure.
7. Procedure‐related adverse events: number, type, and rate of the adverse events that occur during the procedure and during 12 months follow‐up.
8. Patient‐reported outcomes: EQ‐5D‐5L, Specific Symptom Scale Score, and EHRA Score of AF‐related Symptoms.
9. Health Economic units: resource consumption and the associated costs in terms of ablation procedures, follow‐up, management of complications, and health care resources utilization.

### 2.9 Additional analyses

Additional evaluations will be performed. Specifically, the incidence and location of antral low‐voltage activation targets and the rate and location of any PV reconnection after first pass ablation will be evaluated.

### 2.10 Statistical methods and data analysis

Continuous data are expressed as mean ± standard deviation or median values with interquartile range for all the variables collected on the entire population or specific subgroups. Continuous variables will be compared by means of Student *t* test, analysis of variance, or nonparametric test (median test or Mann–Whitney *U* test). Categorical data will be compared by means of the *χ* ^2^ test (Pearson, Yates, or Fisher exact test, as appropriate). Baseline clinical variables, peculiar parameters of the ablation/mapping system, procedural, and postprocedural variables will be tested by regression analysis to evaluate their association with the primary and secondary endpoints. Therefore, independent predictors will be identified by multivariate regression.

Risk estimations (“hazard ratio” 95% confidence interval) will be presented together with the cumulative survival curves (Kaplan–Meier) and the frequency of events (per 100 person‐years) in each group. A specific univariate and multivariate analysis will be carried out to identify the most predictive factors of outcome in a short‐ and long‐term period. A *p* value of 0.05 will be considered statistically significant.

### 2.11 Sample size justification

The DELETE AF study is designed to be observational and comparisons to published data will be done in a post hoc fashion. The sample size requirements for this study are intended to provide adequate power for the analysis of the primary objective.

Assumptions and their rationale:

- 75% Atrial arrhythmias recurrence‐free rate (performance goal set according to published data).^15^, ^16^, ^17^, ^18^
  Both large, multicenter randomized clinical trials^16^, ^17^ and pooled results^15^ that compared the two most common ablation approaches (RF ablation and cryoablation) demonstrated that the efficacy of cryoablation was noninferior to RF ablation at 12 months. In a very recent metanalysis,^18^ the most advanced RF ablation technology was associated with higher freedom from AF/atrial tachycardia at 12 months, compared with conventional ablation catheters in paroxysmal AF patients (75.6% vs. 64.7%; *p* = .015).
- 85% Expected performance in the study group
  In a single‐centre study, Segerson et al.^8^ demonstrated a 90.7% recurrence‐free rate in patients who underwent high‐density mapping (HDM)‐guided PVI and subsequent mapping and ablation of concealed low‐voltage signals through a structured workflow vs. a 73.9% recurrence‐free rate in the group of historical controls who underwent traditional PVI alone. Considering the multicenter setting of the present study, the variable number, and the experience of the operators performing ablation procedures, we set this cutoff to 85%.
- Actual power 90% (*β* level set at 0.1)
  The proportion of patients free from any clinical atrial arrhythmias (primary objective) will be measured in the subgroup of “Treatment” subjects. The number of “Attempt” patients (subjects the procedure is not performed according to the prespecified workflow) is not known. Therefore, the enrollment phase will continue until 200 patients are included in the “Treatment” group (190 patients + 5%, i.e., maximum expected attrition rate during follow‐up), up to a maximum of 300 subjects.

### 2.12 Adverse events management

This study evaluates commercially approved devices within their current labeling, and thus no specific procedures for safety monitoring will be applied in the DELETE AF Study. Centers are to adhere to their IRB reporting requirements and should report incidents with medical devices to the manufacturer or to the National Competent Authority depending on the national practice.

## 3 DISCUSSION

Nowadays, the most commonly used procedure for AF ablation by means of RF technology is a point‐by‐point ablation via a single‐tip catheter combined with a 3D mapping system. RF‐based ablation in conjunction with an electroanatomical mapping system allows a significant reduction of fluoroscopy dosage and provides additional, potentially useful information, such as the LA activation pattern and LA voltage. Beyond that, this technology facilitates an optimized treatment of an additional substrate, non‐PV triggers, or atrial tachycardia.^19^, ^21^

Despite the acute high success rate, the long‐term follow‐up is still characterized by a high number of arrhythmias recurrence mainly related to PV–LA reconnections or non‐PV triggers. Recently, new technologies have allowed an improvement in achieving reproducible and durable PV isolation,^10^, ^22^, ^23^ as well as assessing true PV isolation or residual atrial activity inside the RF lesions.^24^

In a very recent metanalysis,^18^ the use of spring sensor‐irrigated contact force‐sensing catheter was associated with significantly increased odds of the primary endpoint, freedom from AT at 12 months, compared with comparator ablation catheters. Moreover, evidence suggests improved clinical success in paroxysmal AF ablation with stable catheter–tissue contact.^18^ However, a significant limitation of this approach is the absence of tissue feedback during lesion creation, which results in a blunt “one‐size‐fits‐all” approach.^25^ Encouraging results with a novel technology that measures LI through mini‐electrodes on the tip of a dedicated ablation catheter both in an acute setting^9^, ^11^, ^26^ and on arrhythmia recurrence during long‐term follow‐up^10^, ^27^ have been recently reported. However, without intraprocedural guidance regarding where existing lesions are most vulnerable to reconnection, it is difficult to improve on these results.

The Lumipoint^TM^ tool has improved the ability to provide a fast, automated, and comprehensive overview of multielectrode signals and potentially help in the interpretation of complex circuits and low‐voltage electrograms.^28^, ^29^, ^30^ Novel insights from this revolutionary HDM with rapid acquisition of thousands of activation points deepened physiological and pathophysiological understanding of cardiac electrophysiology and arrhythmogenesis. This includes the cardiac conduction system itself and more importantly due to the number of affected patients, the healthy, and diseased working myocardium.^31^ In that sense HDM‐based targeted ablation, in conjunction with pacing maneuvres can be helpful to avoid excessive ablation.^32^ Moreover, HDM has also been found to allow greater precision in the identification of reconnection gaps in PVs; therefore, targeted ablation resulted in lower RF time for PVI.^33^ In addition, the technological advances in cardiac mapping present great opportunities for improving the electroanatomic characterization of low‐voltage activity in tissues such as the PV antra in a faster and more reproducible way.

Segerson et al.^8^ demonstrated in a single‐centre study a 90.7% recurrence‐free vs. 73.9% comparing 150 patients undergoing HDM‐guided PVI and subsequent mapping and ablation of concealed low‐voltage signals (39 redo, 111 de novo) against 452 historical controls undergoing traditional PVI alone. This result represents one of the highest ablation success rates reported in the literature. In their experience, they found a relatively high prevalence of low‐voltage propagation after PVI, similar to that of residual excitability with pace capture studies. They further demonstrated the ease with which high‐resolution mapping can be incorporated in PVI procedures, with no significant effect on procedure time or complication rates.^8^ To date, however, these findings have not yet been demonstrated in a multicentric fashion and are subject to the interpretation and advanced skills of the operators. We designed this study to verify the hypothesis that an improved procedural workflow for catheter ablation, with high‐density mapping system and LI technology to guide the ablation, can improve the 1‐year outcome in patients with paroxysmal AF, without reducing the procedure safety.

## 4 CONCLUSIONS

The DELETE AF trial will provide additional knowledge of long‐term outcomes following a structured ablation workflow, with high‐density mapping and LI technology, in an international multicentric fashion.

## CONFLICTS OF INTEREST

Maurizio Malacrida is an employee of Boston Scientific. The remaining authors have no conflict of interest to declare.

## Data Availability

The data that support the findings of this study are available from the corresponding author upon reasonable request.
